# Reply to ‘Sulfisoxazole does not inhibit the secretion of small extracellular vesicles’

**DOI:** 10.1038/s41467-021-21075-w

**Published:** 2021-02-12

**Authors:** Chan-Hyeong Lee, Ju-Hyeon Bae, Jong-In Kim, Ju-Mi Park, Eun-Ji Choi, Moon-Chang Baek

**Affiliations:** Department of Molecular Medicine, CMRI, Exosome Convergence Research Center (ECRC), School of Medicine, Kyungpook National University, Daegu, Republic of Korea

**Keywords:** Cancer, Cell biology

**Replying to** P. Fonseka et al. *Nature Communications* 10.1038/s41467-021-21074-x (2021)

In the previous paper, we showed various evidences that sulfisoxazole (SFX) inhibits the secretion of small extracellular vesicles (sEVs) from MDA-MB231 human breast cell line^1^. The evidences came from the number of sEVs based on nanoparticle tracking analysis (NTA) results, the protein amount of sEVs, and electron microscopy. In addition, we identified the target of SFX, endothelin receptor A (ETA). Inhibitors of ETA, including zibotentan, BQ123, and PD156707, also inhibited the secretion of sEVs from MDA-MB231 cells. Furthermore, the knockdown of ETA with an ETA short hairpin RNA significantly decreased the secretion of sEVs. Collectively, we showed the inhibition of sEV secretion from MDA-MB231 via ETA through pharmacological and genetic approaches. However, Mathivanan’s group argues that SFX does not inhibit the secretion of sEVs.

In our original publication, we showed that SFX (Sigma, S6377) inhibits the secretion of sEVs from the MDA-MB231 human breast cell line^1^. In addition, two independent researchers again confirmed the original result with a blind test (with SFX from Sigma, 31739). We also show the inhibition effect of sEV secretion from MDA-MB231 cells by two different SFXs (Sigma, 31739, and manufactured SFX) (Fig. 1). Collectively, these results show that SFX does inhibit the secretion of small extracellular vesicles in our experiments.

**Fig. 1:**
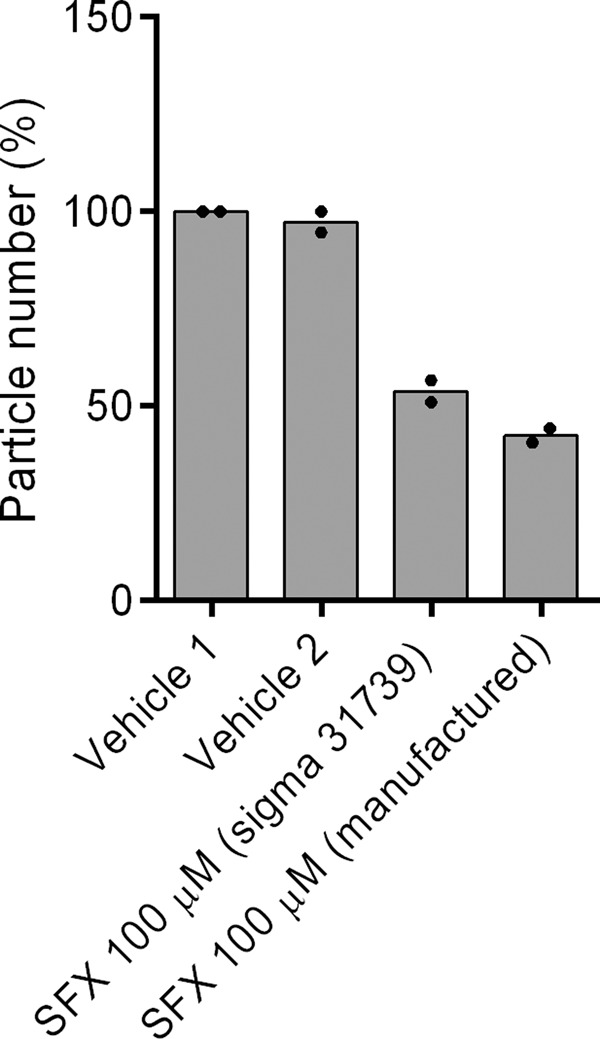
sEV secretion from MDA-MB-231 cells with or without SFX 100 µM (Sigma 31739, and manufactured SFX). All experiments were performed with a blind test (*n* = 2 biologically independent samples).

The protocol we used to separate sEVs is the same as the published manuscript; supernatants obtained from MDA-MB231 cells were serially centrifuged at 300 × *g*/3 min, 2500 × *g*/20 min, and 10,000 × *g*/30 min. In addition, cells were seeded with Dulbecco’s modified Eagle’s medium (DMEM)/high glucose (HyClone, SH30243.01) with 10% fetal bovine serum (FBS) (HyClone, SH30084.03), but it was incubated in DMEM/high glucose without FBS when the cells were treated with SFX.

The two points, we thought, should be essentially considered. First is the cytotoxic effect of SFX due to, probably, an unexpected impurity of SFX from different companies. We used SFX concentrations without cytotoxicity in all experiments. If the drug causes cytotoxicity, the number of secreted sEVs might be increased. Second, large amounts of particles still remain in EV-depleted FBS in our laboratory’s studies. Therefore, it may be difficult to observe the suppression of sEV secretion using EV-depleted FBS.

We uploaded detailed materials to the protocol exchange (https://protocolexchange.researchsquare.com).

## Methods

### Cells and cell culture

MDA-MB231 cells were obtained from the American Type Culture Collection and grown at 37 °C under a humidified atmosphere with 5% CO_2_ and 95% air using DMEM (Hyclone) with 10% FBS (Hyclone) and 1% antibiotic–antimycotic solution (AA, Hyclone). All cell lines were tested for mycoplasma contamination by polymerase chain reaction genotyping.

### Drugs

SFX (31739) was purchased from Sigma-Aldrich. Manufactured SFX was synthesized and provided by Daegu-Gyeongbuk Medical Innovation Foundation, Republic of Korea.

### Isolation of sEVs and analysis

- *Step 1*: Seed the 5 × 10^6^ cells in 150-mm plate (20 ml DMEM with 10% FBS, 1% AA).
- *Step 2*: After 24 h, wash using phosphate-buffered saline (PBS) and then change the media (20 ml DMEM without FBS, AA) with the drug.
- *Step 3*: After 24 h, collect the cell culture supernatant.
- *Step 4*: Centrifuge at 300 × *g*, 4 °C, 3 min and transfer the supernatants to a new tube.
- *Step 5*: Centrifuge at 2500 × *g*, 4 °C, 20 min and transfer the supernatants to a new tube.
- *Step 6*: Centrifuge at 10,000 × *g*, 4 °C, 30 min and transfer the supernatants to a new tube.
- *Step 7*: Filter the supernatants using 0.22-µm syringe filters and transfer the supernatants to a new tube (#344058, Beckman Open-Top Tube).
- Step 8: Ultracentrifuge at 120,000 × *g*, 4 °C, 90 min.
- *Step 9*: Resuspend sEV pellets with 30 ml PBS and centrifuge at 120,000 × *g*, 4 °C, 90 min again.
- *Step 10*: Remove the supernatants and add the 1 ml PBS.
- *Step 11*: After sealing the tube with parafilm, store at 4 °C, 1 h and resuspend the pellet.
- *Step 12*: Transfer the samples to e-tube and analyze using NanoSight and GraphPad Prism.

### Reporting summary

Further information on research design is available in the Nature Research Reporting Summary linked to this article.

## Supplementary information

Reporting Summary

## Data Availability

The detailed protocol has been deposited to the Protocol exchange (10.21203/rs.3.pex-1159/v1). All relevant data of this study are available from the corresponding authors upon reasonable request.

## References

[1] Im EJ (2019). Sulfisoxazole inhibits the secretion of small extracellular vesicles by targeting the endothelin receptor A. Nat. Commun..

